# ‘More than Just a Personal Assistant’: A Qualitative Study Examining the Lived Experiences of Anaesthetic Nurses in Australia

**DOI:** 10.3390/nursrep16050157

**Published:** 2026-05-07

**Authors:** Mary Rose Arcedo, Julie Flynn, Daniel Terry

**Affiliations:** 1 School of Nursing and Midwifery, University of Southern Queensland, Ipswich, QLD 4305, Australia; julie.flynn@unisq.edu.au (J.F.); daniel.terry@unisq.edu.au (D.T.); 2 Institute of Health Research, University of Southern Queensland, Toowoomba, QLD 4305, Australia; 3 Institute of Health and Wellbeing, Federation University, Ballarat, VIC 3350, Australia

**Keywords:** anaesthetic nurse, nurse anaesthetist, perioperative nursing, qualitative research, workplace culture, professional hierarchy, education and training, Australia

## Abstract

**Background/Objectives:** Anaesthetic nurses play a critical role during surgical procedures. However, research focusing on Australian anaesthetic nurses remains limited. While previous studies have identified inconsistencies in anaesthetic nurse education, the everyday experiences of these nurses have not been comprehensively examined. This study aimed to explore the barriers and enablers influencing anaesthetic nursing practice in Australia and to examine anaesthetic nurses’ views on their evolving roles and responsibilities. **Methods:** A hermeneutic phenomenological approach was employed to explore the lived experiences of Australian anaesthetic nurses. Semi-structured interviews were undertaken to enable in-depth exploration of participants’ experiences, thoughts, feelings, and beliefs. Participants were interviewed by telephone, videoconference, or in person. Data were transcribed verbatim into Microsoft Word and analysed using reflexive thematic analysis, informed by Gadamerian hermeneutics. Consolidated Criteria for Reporting Qualitative Research (COREQ) guidelines were followed. **Results:** Four overarching themes were identified, Culture, Education, Leadership, and Institution, each shaping anaesthetic nursing practice in distinct yet interrelated ways, with several subthemes emerging within each category. These interrelated factors contributed to perceptions of being undervalued, restricted career progression, and uncertainty regarding role sustainability. **Conclusions:** The findings highlight the need for enhanced support systems and system-level reform that addresses hierarchical power dynamics alongside standardised, context-specific education and training pathways. Addressing these interconnected issues is essential to better support anaesthetic nurses while ensuring competent, high-quality care is provided. Understanding the structural and cultural concerns underpinning anaesthetic nursing practice may inform the development of coherent curricula, visible nursing leadership, and clearer professional recognition and career pathways.

## 1. Introduction

Anaesthetic nurses work within operating theatres as essential members of the perioperative team, specifically alongside anaesthetists, to provide comprehensive anaesthetic care [1]. They play a critical role in patient advocacy during surgical procedures, particularly when patients are in vulnerable states, thereby ensuring the delivery of safe and compassionate care [2]. In the United States (US), anaesthetic nurses may advance from assisting anaesthetists to practising independently as Certified Registered Nurse Anaesthetists [3,4]. Advanced practice nurse anaesthetists also play key roles in countries such as Sweden, France, and Switzerland. While the scope of practice varies across countries, nurse anaesthetists often administer anaesthesia with autonomy, providing quality, affordable, and accessible care where anaesthetic services might otherwise be unavailable [5,6].

In Australia, anaesthetic nurses work in operating theatres, providing patient care and assisting the anaesthetist. Their primary role is to prepare patients for surgery, assist with anaesthesia, and monitor patients during and after procedures [7]. Without an established advanced practice pathway, anaesthetic nurses in Australia remain limited in their roles [1]. Although Australian anaesthetic nurses demonstrate substantial skill and experience, educational pathways, a lack of structured competency development, and institutional barriers limit their professional progression [1]. Advancing their practice may offer greater support to and accessibility of care in areas with limited healthcare access and to vulnerable populations [8].

Currently, research regarding Australian anaesthetic nurses remains limited, while their roles and educational requirements may not be clearly defined [1]. Given the dearth of research in Australia, further investigation is warranted. While only two studies identified in the literature noted a lack of consistency in anaesthetic nurses’ education across Australia [1,7], the lived experiences and realities of daily practice among Australian anaesthetic nurses have not been explored. To address this gap, this study aims to explore the barriers and enablers in anaesthetic nursing practice in Australia and to examine anaesthetic nurses’ views on their evolving roles and responsibilities. In line with the aim, the study sought to explore anaesthetic nursing practice in Australia, including factors related to patient care, professional development, and the workplace environment, along with what is needed to advance their practice in Australia.

Specifically, the research questions included the following:

1. What are the lived experiences of registered nurses working as anaesthetic nurses as they practice in Australia?
2. What are the key barriers and enablers experienced by anaesthetic nurses that may influence their practice?

By examining the lived experiences of anaesthetic nurses, this study seeks to enhance understanding of how educational preparation and clinical practice are experienced within contemporary anaesthetic settings. It seeks to explore perceived alignments and gaps between existing education and the realities of practice, particularly in relation to role expectations, skill development, and professional confidence. In doing so, the study intends to illuminate how workplace culture, hierarchical structures, and access to relevant education may shape anaesthetic nursing practice. This focus seeks to inform educators, clinical leaders, and policymakers by clarifying areas of concern articulated by anaesthetic nurses themselves and by supporting more contextually responsive insights into education, workforce planning, and professional development.

## 2. Materials and Methods

The study adopted a phenomenological approach to explore the lived experience of anaesthetic nurses in their day-to-day practices, interactions, and clinical environments, and to capture the essence of what it is like to work in this specialised role [9]. To achieve this, the study adhered to the COREQ guidelines.

### 2.1. Participants

Registered nurses working as anaesthetic nurses with at least six months’ experience in different healthcare settings across Australia were included. This was due to their involvement in advanced decision-making, leadership, and a broader range of duties than other perioperative staff [10,11]. There were no limits on where they were working. As such, those working in public or private hospital operating theatres were included, as were those working in procedural and day-surgery centres. There was no geographic limitation across Australia. The approach ensured that the study captured a broad understanding of anaesthetic nurses’ experiences, practice, and challenges across Australia. Enrolled nurses and anaesthetic technicians, although they assist anaesthetists, were excluded due to their distinct roles and responsibilities.

### 2.2. Recruitment

Recruitment was facilitated through purposive and snowball sampling strategies to maximise national reach, with study advertisements disseminated across various private social media pages. For example, major nursing associations such as the Australian College of Nursing distributed the research information details to their membership, direct invitations were sent, and targeted social media were used for recruitment. Although an initial target of at least five anaesthetic nurses from each Australian state and territory (n = 40) was planned to ensure a broad and diverse sample, the final number of participants was guided by availability, participant interest, and data saturation.

Specifically, sampling and data collection continued until informational sufficiency was achieved, whereby no substantially new insights relevant to the study aims were identified in subsequent interviews. During data collection, interviews were reviewed iteratively alongside early analytic engagement, allowing the research team to assess the depth and richness of experiential accounts. The research team met regularly to discuss interviews and preliminary analytic notes as data collection progressed. As the study progressed, participant narratives increasingly confirmed previously identified meanings rather than generating new conceptual understandings. After successive interviews reinforced existing patterns rather than new experiential insights, a collaborative decision was made by the research team to cease recruitment. In keeping with interpretive phenomenological research, saturation was understood not as code repetition, but as sufficient depth of understanding of the phenomenon under study to address the research questions through shared analytic judgement rather than individual assessment [12]. No participants dropped out.

### 2.3. Data Collection

Semi-structured interviews were selected as they enabled in-depth exploration of participants’ experiences, thoughts, feelings, and beliefs [13]. Further, semi-structured interviews can effectively capture nurses’ detailed aspects of practice, including empirical evidence that regular surveys often disregard [14]. Data were collected by one researcher (MR) as part of their doctoral studies and training between April and November 2025, and each session lasted between 30 and 40 min. Participants were unknown to the researchers and were interviewed once by telephone, via video conference technology, or in person, enabling the collection of insights [15]. During the interviews, the researcher (MR) also took notes to capture key points and nonverbal cues. These notes supported the audio recordings and helped the researcher’s reflections [16]. Almost all interviews were audio-recorded with participants’ consent to ensure accuracy. A small number of participants (n = 3) expressed concern about potential professional repercussions and were only willing to participate on the condition that interviews were not audio-recorded. These concerns reflected sensitivities associated with speaking openly about their lived experiences. In these instances, detailed contemporaneous notes were taken during and immediately following the interviews to capture participant accounts as accurately as possible.

### 2.4. Data Collection Tool

An interview guide was developed after reviewing relevant literature and consulting the research team. Questions were tailored to address the main research questions and piloted with three (n = 3) Anaesthetic nurses to ensure face and content validity, thereby achieving rigour. Consistent with qualitative methodological guidance, three participants were considered sufficient for this purpose, as pilot interviews in qualitative research commonly involve a small number of participants to assess clarity, relevance, and alignment with study aims. Feedback from the pilot interviews resulted in minor refinements to question wording and sequencing, after which the interview guide was finalised [17,18].

The final questions encouraged participants to share detailed experiences, perceptions, and challenges they were experiencing. Interview questions included questions, such as ‘Tell me about your nursing story, for example, how long have you been in anaesthetic nursing, and how did you get into this specialty’, ‘What parts of anaesthetic nursing are most rewarding’, and ‘What parts are the most challenging’ (Appendix A). In addition, probing questions helped clarify and expand on responses. Demographic details such as age, gender, years of experience in anaesthetic nursing, and education were also collected to contextualise the findings.

### 2.5. Data Analysis

Data were transcribed verbatim into Microsoft Word (Version 2510, Build 19328.20244, Redmond, WA, USA) following each interview, including those conducted via videoconferencing. The transcripts were cleaned and reviewed for accuracy, and participants were invited to review and confirm the correctness of their transcripts. Transcripts were labelled according to the order in which participants were recruited (e.g., Participant 1 (P1), P2, P3).

This study adopted an interpretive (hermeneutic) phenomenological approach informed by Gadamer’s philosophical hermeneutics to explore the lived experiences of anaesthetic nurses in their day-to-day practices, interactions, and clinical environments [19]. Hermeneutic phenomenology is concerned with understanding how meaning is constructed through individuals’ experiences, shaped by social, historical, and professional contexts [19]. This interpretive approach acknowledges that understanding emerges through interpretation and dialogue between the participant accounts and the researchers’ situated perspectives [19].

Consistent with Gadamerian hermeneutics, this study adopted an interpretive approach in which understanding was not sought through bracketing pre-understandings in pursuit of description, but through reflexive engagement with participants’ accounts to interpret how meanings are formed and understood in practice. Knowledge was conceptualised as constructed and evolving through an iterative process of engagement with the data, characterised by movement between individual narratives and the dataset as a whole.

Data were analysed using reflexive thematic analysis as described by Braun and Clarke [20], situated within this interpretive phenomenological framework informed by Gadamerian hermeneutics. Thematic analysis was selected as a methodologically flexible analytic approach capable of supporting in-depth interpretation of lived experience while remaining attentive to context, language, and meaning. Within this hermeneutic orientation, themes were not treated as objective entities that ‘emerged’ from the data, but as interpretive patterns of meaning developed through a dialogical and iterative engagement between the researchers and participants’ accounts. The integration of hermeneutic phenomenology and reflexive thematic analysis enabled an interpretive account of anaesthetic nurses’ experiences that emphasised meaning-making rather than description, aligning method, philosophy, and analytic aims.

Consistent with hermeneutic phenomenology, the analytic process did not involve bracketing researcher pre-understandings. Instead, reflexivity was employed to critically examine how researchers’ clinical, professional, and theoretical backgrounds shaped interpretation. Understanding was conceptualised as dynamic and evolving, developed through movement between individual narratives and the dataset as a whole. Microsoft Word, butchers’ paper, and sticky notes were used to facilitate data analysis.

The six phases of Thematic Analysis of Braun and Clarke [20] were undertaken. For example, an analysis commenced with familiarisation, in which the whole research team read and re-read the interview transcripts to develop an initial understanding of the data. This immersive engagement allowed researchers to attune to both explicit descriptions and the underlying meanings conveyed by participants, reflecting the interpretive movement between the parts and the whole that is central to hermeneutic inquiry.

Initial codes were then generated by identifying meaningful features across the dataset and collating significant excerpts related to anaesthetic nursing practice, including challenges, supports, contextual influences, and interpersonal dynamics. These initial codes were data-driven and closely reflected participants’ language and accounts of their lived experiences. Coding was understood as a moment of interpretive understanding, shaped by the researchers’ evolving engagement with the text. All three members of the research team examined transcripts, with codes discussed and refined collaboratively to ensure analytic coherence.

As analysis progressed, a hierarchical coding tree was developed to organise the data and support transparent interpretation. Related initial codes were clustered into subthemes based on conceptual similarity and shared meaning, allowing patterns across individual accounts to be identified while remaining grounded in the data. Subthemes were subsequently synthesised into broader thematic categories, forming a structured analytic progression from initial codes to interpretive themes. The initial codes were grouped into themes, and team members (M.A., J.F., D.T.) collectively sorted coded data into preliminary thematic categories, discussed overlaps, distinctions, and points of interpretive tension, and refined the developing coding tree through iterative dialogue. These discussions produced a thematic map that represented the developing structure of the analysis.

Themes were continually refined to ensure that they were internally coherent, consistent with the coded extracts, and meaningful in relation to the entire dataset. Themes were subsequently defined and named to capture their central organising concepts, with participant excerpts selected to enhance authenticity and confirmability. The final reporting phase involved selecting illustrative quotations, conducting a final interpretive analysis of each theme, and integrating findings with relevant literature to produce a comprehensive account of the lived experience of anaesthetic nurses in Australia. This methodological approach supported a transparent and iterative interpretive process, aligning with phenomenological inquiry while minimising researcher bias and reinforcing trustworthiness [20].

To ensure rigour, several strategies were employed, including peer debriefing, reflexivity, and checking participant transcripts. Peer debriefing occurred through regular meetings between the lead researcher (M.A.) and team members (J.F. and D.T.), who provided critical feedback on analytic decisions, challenged assumptions, and supported deeper examination of emerging interpretations. These discussions strengthened the credibility and robustness of the analysis.

The research team included experienced qualitative researchers with backgrounds in nursing education and clinical practice. The lead researcher (M.A.) is a female anaesthetic nurse, which may have influenced data generation and interpretation. In hermeneutic phenomenology, the researcher’s pre-understandings cannot be bracketed out entirely; rather, they are acknowledged as integral to the interpretive process [19]; however, reflexive practices were used to mitigate the potential impact of bias. Reflexive journals were maintained throughout data collection and analysis to document assumptions, emotional responses, and interpretive decisions. Reflexive writing enhanced self-awareness, transparency, and critical reflection, supporting thoughtful analytic choices [21].

Member checking was undertaken by inviting participants to review their interview transcripts to ensure interpretive accuracy and provide opportunities for clarification. Although not always required in Gadamerian Hermeneutics, in which understanding is viewed as dynamic and situated, member checking was incorporated to enhance authenticity, foster collaboration, and confirm that participants’ voices were accurately represented [22]. It also ensured that participants had the opportunity to add or remove any data they had suggested not be included. No participants offered amendments to their transcripts. Collectively, these approaches ensured the credibility, dependability, and confirmability of the study, producing an analysis firmly centred on the lived experiences and perspectives of anaesthetic nurses.

### 2.6. Reflexivity and Researcher Positionality

Reflexivity refers to the ongoing critical examination of how researchers’ positions shape interpretation; reflexive practice involved deliberate strategies such as journaling and peer debriefing, while reflexive writing was used to document and interrogate assumptions and interpretive decisions across the research process [23]. Occupying the dual role of anaesthetic nurse and researcher positioned the lead researcher (MA) as an insider to the professional group under study. This insider status influenced recruitment by enabling access through established professional networks, shared language, and cultural familiarity within the perioperative environment. Consistent with previous research, this embeddedness fostered trust, reduced scepticism about research participation, and reassured participants of the study’s relevance to their everyday clinical realities [24,25,26]. Operating theatre staff are often reluctant to engage with external research due to time pressures, confidentiality concerns, and perceived disconnection between external investigators and clinical practice; insider status was therefore critical in facilitating meaningful engagement and participation [27,28,29].

Shared professional identity also shaped the data collection process. While participants occasionally appeared to assume a degree of common understanding with the researcher, this was actively addressed during interviews. Rather than confirming or endorsing taken-for-granted meanings, the interviewer intentionally prompted participants to elaborate on their experiences and interpretations (e.g., “Can you describe what that meant for you?”). This strategy disrupted assumptions of shared understanding and encouraged participants to articulate experiences that might otherwise have remained implicit.

From a hermeneutic phenomenological perspective informed by Gadamer, the lead researcher’s pre-understandings were recognised not as biases to be bracketed out, but as integral to the interpretive process [19]. Reflexivity was therefore employed to critically examine how professional knowledge, expectations, and emotional responses shaped engagement with the data. Reflexive journaling was maintained throughout recruitment, data collection, and analysis to document assumptions, moments of resonance, and interpretive tensions, particularly when participants’ accounts challenged or contradicted the researcher’s expectations.

These reflexive observations were systematically discussed with the co-researchers (J.F. and D.T.) during peer debriefing sessions. This process provided analytic distance, supported questioning of taken-for-granted interpretations, and ensured that insider knowledge functioned as an interpretive resource rather than an unexamined authority. Through this dialogical and reflexive process, controversial assumptions and interpretive uncertainties were explicitly surfaced and incorporated into the ongoing analysis.

## 3. Results

Overall, n = 31 anaesthetic nurses were interviewed and were predominantly female, comprising 77.3% (n = 24) of the cohort. More than three-quarters of participants were working as anaesthetic nurses for more than six years (n = 22), half were working in private health services (n = 16), while more than half were also from Queensland (n = 17). Lastly, among all participants, just under 50% had undertaken post-graduate education associated with anaesthetic nursing (n = 14) (Table 1).

**Figure 1:**
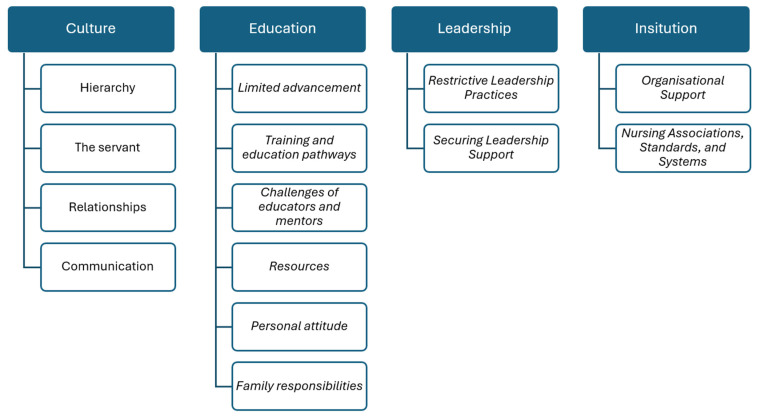
Themes and subthemes pertaining to anaesthetic nursing data.

Within the data, four overarching themes were identified—Culture, Education, Leadership, and Institution—each shaping anaesthetic nursing practice in distinct yet interrelated ways. Several subthemes were identified within each theme, further illustrating the nuanced experiences described by participants. These themes and subthemes are presented below and illustrated in Figure 1. Each will be discussed in detail.

### 3.1. Culture

The first theme was cultural factors, identified as a prevalent barrier in anaesthetic nursing practice, with nurses consistently emphasising how entrenched workplace norms influence daily experiences. For example, the culture of organisations consisted of informal norms, unwritten traditions, and shared values that guided collective behaviour within clinical teams. As such, across various clinical settings, nurses reported a culture characterised by subordination, hierarchical structures, ineffective communication, and strained interprofessional relationships. These cultural dynamics were observed to undermine professional identity, limit autonomy, and adversely affect team cohesion. Four subthemes, which include hierarchy, the servant, relationship, and communication, are discussed in detail.

#### 3.1.1. Hierarchy

Nurses consistently described the operating theatre as a highly hierarchical environment in which anaesthetists hold dominant authority. Nurses reported that this hierarchy frequently manifested through behaviours that dismissed nursing expertise, limited nurses’ influence in clinical processes, and reinforced power imbalances within the anaesthetic team. Such dynamics contributed to a workplace culture in which nurses felt their knowledge and professional judgement were undervalued, particularly when anaesthetists disregarded established protocols or excluded nurses from clinical discussions.

Nurses explained that navigating these hierarchical relationships often required significant emotional regulation and diplomacy. Rather than challenging inappropriate behaviour or questioning clinical decisions, nurses described consciously accommodating anaesthetists’ preferences and temperaments to maintain workflow and prioritise patient safety. This highlighted how anaesthetic nurses manage interpersonal tension, further reinforcing nurses’ subordinate positioning and constrained opportunities for assertive professional engagement. One nurse described anaesthetists “treat you like you don’t know anything… [and]… don’t respect you” (P28), suggesting how hierarchical dominance translated into everyday experiences of professional disregard.

Despite these challenges, nurses also acknowledged variability in individual working relationships. Some described positive, collaborative interactions with anaesthetists, noting that not all anaesthetists engaged in dominant or dismissive behaviours. However, nurses emphasised that such experiences depended on individual attitudes rather than on systemic cultural change, stating that hierarchical dominance remained a persistent and overarching feature of anaesthetic practice.

#### 3.1.2. The Servant

Moving beyond the hierarchy, one recurring sub-theme among nurses was a strong sense of being undervalued and overlooked within the anaesthetic team, with anaesthetic nurses frequently perceived as assistants rather than autonomous professionals. Nurses described how their role was routinely reduced to task-based support for anaesthetists, obscuring their responsibilities for patient assessment, safety, and clinical judgement. One nurse expressed this experience clearly:

*Anaesthetic nursing is all over the place, which is how I think the role has become. It’s more of a servant to the anaesthetist than to the patients. It’s a strange job; I didn’t go into nursing to do that.* (P10)

Nurses explained they were overlooked through everyday practices in which their knowledge and expertise were neither sought nor acknowledged. Although they possessed formal training and substantial clinical experience, their contributions were often limited to basic supportive tasks. At the same time, their role in patient monitoring, risk identification, and perioperative decision-making remained largely invisible. This lack of recognition was further described by limited autonomy, minimal involvement in clinical discussions, and a perceived lack of trust from anaesthetists. Consequently, nurses reported a diminished professional identity and a workplace culture in which their skills were underutilised and their clinical value unrecognised. This feeling of invisibility and lack of recognition was captured by one of the anaesthetic nurses’ comments, who said: “We are not recognised and valued as a valuable part of the anaesthetic team” (P3). Despite these experiences, nurses consistently emphasised that anaesthetic nursing extends beyond assistance and requires specialised knowledge and clinical judgement. They expressed a strong desire for greater recognition and respect, highlighting the need to reframe anaesthetic nursing as a distinct and essential professional role within the anaesthetic team.

#### 3.1.3. Relationships

In the operating theatre, the relationship between anaesthetic nurses and anaesthetists was also highlighted as challenging. The relational dissonance between anaesthetists and anaesthetic nurses creates a difficult atmosphere, resulting in low morale amongst anaesthetic nurses. This perception led nurses to feel degraded in their professional environment. They expressed that, knowing anaesthetists often view them as less knowledgeable, they experience feelings of being looked down upon and undervalued, with one nurse stating, “Anaesthetic doctors treat you like you do not know anything, the… doctors know everything nurses know nothing!” (P28). These perceptions do not just harm their relationship with anaesthetists but also impact their interactions with the entire theatre staff.

Nurses described knowing they are belittled creates a sense of emotional self-protection, leading them to limit interactions with anaesthetists to only essential matters related to patient care. This avoidance, in turn, further undermined communication and collaboration within the team. Nurses reported that the quality of these relationships directly influenced their daily work, professional confidence, and access to informal learning opportunities. As one participant explained, “Not having a good relationship with anaesthetic doctors cuts [reduces] many opportunities [in the workplace]” (P-27), including educational opportunities. Poor relationships with anaesthetists were described as creating an environment in which nurses felt hesitant or fearful to ask questions, even in situations that offered valuable learning opportunities.

#### 3.1.4. Communication

The fourth subtheme identified was communication, where nurses highlighted poor communication and inadequate teamwork as significant cultural challenges within anaesthetic nursing. Several nurses reported that interactions between anaesthetic nurses and anaesthetists were frequently limited or ineffective, thereby complicating daily operations, leading to frustration in the operating theatre.

*No good communication between anaesthetic doctors and anaesthetic nurses, yes… we don’t have solid knowledge as the anaesthetist, that’s probably why they do not see the need to communicate to us.* (P26)

This was further explained by another nurse who stated, “communication is a barrier in anaesthetic nursing practice, and teamwork is also a big concern” (P21). Nurses reported ongoing challenges with communication and teamwork within anaesthetic nursing.

### 3.2. Education

The second theme was education being a challenge, with several sub-themes emerging, including limited clinical and career advancement opportunities, heavy workloads that affect direct patient care, and persistent gaps in structured training pathways. Issues such as the lack of standardised education and insufficient resources were emphasised, highlighting the need for a more cohesive, well-supported approach to anaesthetic nursing education.

#### 3.2.1. Limited Advancement

Nurses consistently expressed frustration over the lack of structured career pathways within anaesthetic nursing. Anaesthetic nurses reported a strong sense that advancement opportunities are limited, creating a ‘low ceiling’ for professional growth. This limitation, as identified by nurses, not only impacted individual motivation but also contributed to role ambiguity and stagnation within the specialty. As one nurse highlighted, “In perioperative, specifically anaesthetic nurses, there is no higher level, no career progression” (P16).

Nurses described career pathways as a key factor influencing both entry into and retention within anaesthetic nursing. For example, nurses who transitioned from recovery and ward settings reported being attracted to anaesthetic nursing because of expectations of exposure to advanced equipment, opportunities to develop new technical skills, and the perceived excitement of working in a theatre environment. However, after commencing work in anaesthetic, the nurses described disappointment with the role, reporting that daily practice primarily involved equipment preparation and assisting anaesthetists, with limited patient interaction and minimal opportunities for independent nursing practice or skill development. A central concern raised was the absence of a clear career pathway, with nurses noting that higher qualifications often led to clinical nursing roles. A senior anaesthetic nurse stated, “If you want registered nurses to become anaesthetic nurses, there has to be advanced practice, a career pathway” (P16). Nurses expressed a desire to reconsider their future in anaesthetic nursing.

#### 3.2.2. Training and Education Pathways

In addition to limited career opportunities, nurses collectively stated that their training within the health system was insufficient. This was perceived as a barrier to enhancing their capacity to practice at the top of their scope. Some nurses took the initiative to pursue their own training opportunities, recognising the importance of continuous professional development. Others reported that pursuing further education not only enhanced their knowledge and skills but also improved patient care and contributed positively to the broader healthcare system. Another senior anaesthetic nurse stated, “more knowledge is never a burden…better for you, for the patient, and the system in general. Better for anaesthetic nurses” (P2). Overall, limited education was identified as a significant barrier to professional growth, affecting their confidence, skills, and practice.

Anaesthetic nurses also noted a lack of standard university-based education for anaesthetic nursing. Many expressed frustrations over the absence of a clearly defined educational pathway for anaesthetic nursing practice. They reported significant difficulty accessing education directly relevant to their roles, especially in critical clinical areas such as airway management, respiratory care, and medication administration. Although some nurses had completed postgraduate qualifications, they felt these programmes were not tailored to anaesthetic nursing and provided limited practical value for their clinical work. Several participants described their postgraduate qualifications as merely a piece of paper, rather than as meaningful preparation for the realities of anaesthetic nursing practice.

Anaesthetic Nurses strongly supported a nationally recognised training pathway and stressed the need for a standard level of knowledge and education to ensure consistency, safety, and professional credibility. A highly experienced anaesthetic nurse stated:

*I wish there were one standard program… The biggest barrier is the lack of standardised training… The lack of a standardised training program means that there is no base scope for anaesthetic nursing.* (P6)

The lack of a national framework was identified as limiting recognition, mobility, and career progression among anaesthetic nurses. Overall, nurses viewed structured university-led education as essential to strengthening entry-level preparation, improving consistency in practice, and advancing anaesthetic nursing as a recognised specialty.

#### 3.2.3. Challenges of Educators and Mentors

Beyond the lack of formal education, nurses reported serious concerns about who is responsible for educating and mentoring anaesthetic nurses and whether those educators and mentors are adequately prepared for these roles. Anaesthetic nurses described a disconnect between education and clinical skills, expressing frustration that many educators lacked a background in anaesthetics, while others had clinical knowledge but limited teaching ability. One experienced anaesthetic nurse stated.

*The educators… they could have knowledge but not be good at teaching… or… they are good at teaching but have no knowledge or background in anaesthetics. Lack of preceptorship training. It is important to be good for both of those… and they should have current practice as an education coach.* (P31)

Anaesthetic nurses also reported being assigned as preceptors without formal training. One nurse articulated, “I precept graduate nurses, I don’t even have training as a preceptor… anaesthetic nurses who just finished grad program, they become preceptors straight away” (P14). Nurses are becoming preceptors despite their own limited experience, creating a cycle in which underprepared nurses train others. They indicated that reliance on the buddy system and short rotations under non-anaesthetic educators did not provide adequate clinical depth. Some nurses reported that they particularly valued the involvement of anaesthetists as mentors, believing that interprofessional education enhanced their learning and strengthened teamwork. Nurses also noted this concern, emphasising that newly graduated nurses who have completed a year of perioperative nursing training are now assigned as preceptors, thereby reinforcing the challenges they face. Anaesthetic nurses expressed the need for better-prepared educators, structured preceptor training, and stronger collaboration with anaesthetists.

#### 3.2.4. Resources

In addition to challenges related to training availability and the quality of educators and mentors, nurses consistently identified resource constraints as a significant barrier to accessing education and professional development. Heavy workloads and chronic understaffing were reported to limit nurses’ ability to take study leave, even when relevant education opportunities were available. One nurse explained, “Because of the shortage of staff, you will not be allowed to take leave to attend education and training” (P14). Nurses described this as ‘routine’, with workforce demands frequently taking precedence over educational needs. Anaesthetic nurses also reported difficulties accessing education programmes that were relevant to anaesthetic nursing practice. Available courses were described as difficult to access, poorly aligned with clinical practice, and constrained by organisational pressures, reducing the feasibility of undertaking further study while working in anaesthetic settings.

Financial constraints further limited access to education and professional development. Nurses consistently reported that the high cost of university courses and external training restricted their ability to pursue advanced learning. One nurse stated, “Courses are expensive… I can’t afford them” (P2). While some nurses reported that employer-sponsored subsidies, more commonly in private hospitals, partially reduced the financial burden, others noted that such support was inconsistent or unavailable. Overall, nurses described staffing shortages, limited study leave, and financial costs as interconnected resource barriers that restricted access to education and professional development within anaesthetic nursing practice.

#### 3.2.5. Personal Attitude

Anaesthetic nurses expressed differing views regarding the role of individual attitudes in advancing anaesthetic nursing practice. While some nurses attributed limited role progression to broader structural and organisational barriers, others perceived that attitudes within the nursing workforce also influenced engagement with professional development and role expansion. Many described a culture of complacency, in which some colleagues become overly comfortable with routine tasks and reluctant to take on higher-level responsibilities. One nurse noted that avoiding initiative leads to stagnation, suggesting “If you can’t initiate too many things, it means you don’t need to do critical thinking… you just become lazy and get so used to the system” (P26). Fear, self-doubt, and lack of confidence were also identified as significant obstacles. Several participants noted that this reluctance is compounded by a preference for performing fundamental or basic tasks, resistance to further education, and the belief that specific advanced responsibilities should remain the domain of doctors.

Several nurses also described the belief that a reluctance to pursue expanded responsibilities or further education existed within the profession. For example, an anaesthetic nurse, with more than 20 years of experience working in the role, stated, “Honestly, the biggest barriers in advancing anaesthetic nursing practice are the anaesthetic nurses themselves” (P23). These views were often expressed alongside accounts of long-standing role constraints and limited professional autonomy. Nurses described how confidence, motivation, and willingness to pursue expanded roles were shaped by their everyday experiences within anaesthetic settings, including repeated exposure to hierarchical decision-making and restricted opportunities for advancement.

#### 3.2.6. Family Responsibilities

Lastly, nurses described family responsibilities as a significant barrier to advancing anaesthetic nursing practice, with several highlighting that balancing family life, demanding work schedules, and the need to develop new clinical skills were ongoing challenges. Many nurses expressed difficulty in balancing early shifts, long hours, and family commitments, particularly for those with young children. A junior anaesthetic nurse explained, “I have two young children… working as an anaesthetic nurse requires starting early… pursuing further studies would make it even more challenging” (P27). Others highlighted the emotional and physical strain of irregular hours, noting that shift work is challenging and can lead to burnout among anaesthetic nurses. The competing demands of family life, personal responsibilities, and economic pressures were described as intensifying the difficulty of advancing anaesthetic nursing practice.

### 3.3. Leadership

Leadership in nursing that encompasses formal managerial behaviours, formal norms, enforcement, and governance systems were often perceived as a barrier within anaesthetic nursing practice. As the third pillar of this study, leadership is examined in terms of how formal hierarchical structures and insufficient advocacy for anaesthetic nurses at the executive level restrict support and innovation, thereby confining nursing practice to rigid, traditional workplace environments.

#### 3.3.1. Restrictive Leadership Practices

Nurses identified disempowering leadership practices as a significant barrier to advancing anaesthetic nursing, describing a culture characterised by limited support, micromanagement, and exclusion from meaningful decision-making. Anaesthetic nurses described leadership behaviours that were perceived to restrict autonomy and critical thinking, with one nurse explaining that they were often “just told to do and expected to have no opinion… and if you question anything you can be disciplined” (P2). Anaesthetic nurses also highlighted concerns about leaders lacking clinical insight, prioritising hospital revenue over nursing issues, and maintaining fear-based or territorial attitudes that suppressed innovation. Nurses expressed dissatisfaction and felt unsupported when leaders had no background in anaesthesia, noting that this contributed to stagnation and resistance to recognising the complexity of anaesthetic nursing roles.

#### 3.3.2. Securing Leadership Support

Anaesthetic nurses with more than ten years of clinical experience reported that securing sustained leadership support for role development was challenging, and that leadership withdrawal led to immediate setbacks in the advancement of anaesthetic nursing roles. Nurses described how programmes collapsed when key champions left, reflecting the fragility of progress in the absence of strong leaders. One experienced nurse described:

*When a supportive leader retired, the program advancing the nursing practice eroded… the new director of nursing hoped we would just leave the role or retire because it was a complicated issue.* (P23)

Nurses frequently described leadership as demonstrating concerns about legal ramifications, maintaining conservative positions on scope-of-practice expansion, and, at times, displaying tall poppy syndrome. Anaesthetic nurses identified these behaviours as discouraging to those aspiring to develop advanced practice skills. Discouragement from senior staff underscored how leadership resistance undermined confidence and hindered professional growth in advanced anaesthetic nursing roles, as reported by anaesthetic nurses.

### 3.4. Institution

The final theme examines how nursing standards fail to accommodate the dynamic, technologically advanced environment in which anaesthetic nurses operate. This is within the context of how institutions view nurses as a budgetary cost rather than valuable assets, particularly in profit-oriented settings.

#### 3.4.1. Organisational Support

Nurses described institutional conditions, particularly management practices, staffing levels, workload demands, and organisational priorities, as significant barriers to anaesthetic nursing practice. They reported high patient turnover, chronic understaffing, limited breaks, and poorly organised workflows, all of which contributed to fatigue, stress, and feelings of being undervalued. As one nurse explained, “The role is stressful because there is just a high turnover of patients and we are understaffed” (P2). Several nurses also emphasised the lack of meaningful support from hospital executives and managers. Hospital management often did not understand the complexity of anaesthetic nursing care for patients, and, as executive management, they failed to address workplace abuse and unsafe workloads. Nurses stated that individuals in executive or leadership positions do not realise that professional development is essential for nurses, leading to the loss of advanced practice opportunities and, in turn, to some nurses becoming ‘deskilled’. Nurses described working in conditions of high demand, limited resources, and limited leadership insight, which they associated with challenges to safe and sustainable anaesthetic nursing practice.

#### 3.4.2. Nursing Associations, Standards, and Systems

Anaesthetic nurses also identified broader institutional structures, such as professional bodies, legislation, and standards frameworks, as contributing to barriers to the advancement of anaesthetic nursing practice. Many expressed that existing guidelines lacked specificity and did not adequately represent the specialised role of anaesthetic nurses. One anaesthetic nurse noted that “legislation, nurses’ organisation, credentialing frameworks are not really supporting anaesthetic nurses to advance their practice” (P17). Others stressed the need for a dedicated anaesthetic nursing body to develop standards that accurately align with clinical realities and to collaborate more effectively with anaesthetic nurses. Nurses reported that current standards prioritise compliance over providing substantive professional support, making it difficult for them to pursue advanced practice roles. They described how these systemic limitations within regulatory and professional frameworks were identified as a cause of the ongoing stagnation in the development of anaesthetic nursing.

## 4. Discussion

This study provides an exploratory account of how anaesthetic nurses describe their roles, challenges, and professional contexts. Given the qualitative design and the concentration of participants from one state, the findings are intended to offer illustrative insight into lived experiences rather than to provide national representativeness of Australian anaesthetic care. Nevertheless, the challenges experienced among anaesthetic nurses are more complex than earlier reports have suggested. Previous Australian studies have shown a connection between medical dominance and changes in anaesthesia roles [1,7]. However, these perspectives may have overlooked certain socio-cultural and educational factors as noted in this research. Importantly, the findings of this study indicate that these factors are not discrete, but interrelated and mutually reinforcing, with medical hierarchy functioning as a central organising influence across multiple aspects of anaesthetic nursing practice. Culture was identified in this study as a barrier to anaesthetic nursing practice. Power imbalances in anaesthetic nursing are best understood as manifestations of deeply embedded professional territoriality within a longstanding medical culture that differentiates between superior and subordinate roles. Reports of nurses experiencing professional disrespect in interactions with doctors are well-documented and reflect a persistent hierarchical structure that may continue to perpetuate such dynamics [30].

Some participants described attitudes within the anaesthetic nursing workforce as contributing to limited role advancement. While these accounts were reported verbatim in the Results, they warrant careful interpretation. Such views frequently emerged alongside descriptions of long-standing hierarchical structures, restricted professional autonomy, and limited opportunities for progression. When considered within this context, perceptions of individual reluctance or diminished motivation are better understood as shaped by repeated exposure to structural constraints rather than as evidence of inherent personal deficits. Rather than attributing marginalisation to individuals, these findings underscore how professional attitudes are influenced by the conditions in which anaesthetic nurses practice [31]. This highlights how cultural and hierarchical environments may shape not only external constraints but also internalised perceptions of role possibility, confidence, and leadership readiness.

This jurisdictional tension extends beyond personal ego and represents an ongoing struggle for legitimacy within a healthcare system that remains predominantly medical-centric [32]. The relationship between anaesthetic nurses and anaesthetists is frequently described as strained, with some doctors perceived as disrespectful or unwilling to communicate, which further limits nurses’ opportunities for professional development. Such relational dynamics may have a downstream effect, influencing access to informal learning, mentorship, and support for role expansion. Hierarchical power dynamics serve as barriers to role advancement, and professional boundaries are reinforced by the expectation that only doctors should administer anaesthesia [32]. The issue of physicians acting as barriers to the advancement of nursing practice is prevalent [33]. Extant literature suggests that nurse endoscopists, as an example, encounter similar challenges [34]. Advancing the role of endoscopy nurses is a solution to reduce patient waiting lists for endoscopic procedures. However, the Gastroenterologist asserts that only the medical practitioner model ensures safe and competent endoscopy and advocates funding endoscopy physician training rather than nurse training [35,36], emphasising that physicians are more qualified to manage complex cases [37].

A study conducted in the United Kingdom (UK) found that at least one-third of theatre nurses have experienced relationship issues with doctors, including dismissive or aggressive communication [38]. In contrast, this issue was not observed in Norway, where doctors are reported to be supportive of nurses, although the study was not directly related to theatre or anaesthetic nursing [39]. Norwegian doctors emphasised competent care would be impossible without nurses’ support, highlighting that team support facilitates the resolution of challenging clinical cases. This collaborative approach is believed to enhance team functionality and productivity [39]. These contrasts suggest that hierarchical dominance is not inevitable but is shaped by context, reinforcing the importance of organisational and cultural structures in determining how professional relationships develop. Although these roles differ between countries, it is vital to note that these experiences are occurring across similar roles within the perioperative space. However, to gain a comprehensive understanding of these professional tensions within Australia, further research is needed to explore anaesthetists’ perspectives and experiences regarding the anaesthetic nursing role, which was outside of the scope of this study.

While workplace culture shapes the daily environment, education constitutes a second critical pillar underlying the current crisis in anaesthetic nursing; the two fuel each other. Nurses’ accounts indicate that educational gaps are closely intertwined with hierarchical role positioning, where reduced recognition of anaesthetic nursing expertise contributes to limited investment in specialised education and training pathways. The study found the ‘invisible’ nature of the role is intensified by a persistent lack of structured educational pathways, which hinders anaesthetic nurses from articulating their specialist value. Concerns identified by Ireland and Osborne [7] regarding a lack of standardised training, which continues to serve as a primary barrier for Australian anaesthetic nurses. Nurses described not only the absence of mandatory national qualifications but also that university postgraduate courses were often generic and failed to address the discipline’s specialised technical requirements. Ireland and Osborne [7], who emphasised that there is a lack of consistent, mandatory national education standards for anaesthetic nursing. Michaels and Foran [1] argued that the role of anaesthetic nurses in Australia has reached a professional impasse due to the absence of recognised advanced clinical practice pathways. Together, these findings suggest a cyclical relationship in which limited education reinforces perceived subordinate roles, which in turn constrain advocacy for educational advancement. The issue extends beyond standard training and education for anaesthetic nurses and is widespread within the nursing profession [30]. In Australia, the lack of a nationally standardised training and credentialing framework significantly hinders the advancement of nursing in specialty areas [40,41,42,43].

In the context of anaesthetic nurses, limited specialised knowledge often leads to these roles being perceived as subordinate, with responsibilities primarily restricted to preparation and support rather than to substantive clinical input. This perception further limits nurses’ influence in clinical decision-making and leadership processes. Nurses reported feelings of marginalisation and have noted that their patient advocacy is frequently undervalued by theatre staff. Comparable challenges have been documented among other nurses, such as those who provide care in the community. Primary health or community nurses have specialised training and experience; however, they still encounter being belittled, ignored, and dismissed by medical staff, which has ongoing impacts, such as moral stress [27]. In contrast, nurses employed in hospitals with nurse-led clinics in Henan province, China, reported that their profession as registered nurses is valued and recognised. In these clinical settings, nurses operate in non-technical capacities and are still regarded as essential members of the health care team, deeply respected and never overlooked [44].

Resource constraints were another concern, especially understaffing and high course costs, which remain significant issues for anaesthetic nurses. Managerial priorities often prioritise immediate staffing needs over long-term professional development [45]. These resource limitations may be compounded by hierarchical decision-making structures, in which nursing education is deprioritised relative to organisational and medical imperatives. Additionally, anaesthetic nurses reported concerns regarding anaesthetic educators, who were often perceived as lacking both clinical expertise and effective teaching skills. Most theatre educators are not anaesthetic nurses, a key reason for their limited ability to support anaesthetic nurses in education and training. Hakim [46] has indicated that a high proportion of challenges in clinical nursing education is attributable to factors associated with clinical educators who lack skills, along with a lack of pedagogical training. Despite these concerns, an inconsistent approach to recruiting nurse educators contributes to performance challenges [47]. This issue is often overlooked, as it is assumed that nurses transitioning into educator roles will naturally adapt and eventually fulfil their responsibilities effectively within the hospital setting [47]. At times, this may occur without considering the necessary academic qualifications, formal support, or standardised recruitment processes, and may lead to performance challenges or professional development needs inadvertently being overlooked or misunderstood [48].

Leadership in nursing is also a determining factor of both the quality of patient care and the work environment for nursing staff. Nurse leaders experience moral distress when faced with conflicting demands between organisational accountability and their responsibility to maintain staff morale. In anaesthetic nursing, effective leadership is particularly essential given the high-stakes responsibilities and the rapid pace of clinical practice. However, nurses’ accounts suggest that leadership capacity is constrained by the same hierarchical structures that limit education and role recognition, positioning nurse leaders in mediating rather than transformative roles. Nurse leaders are required to balance advocacy for staff well-being with the achievement of organisational objectives [49]. Persistent tension may lead to the neglect of nurses’ needs, negatively affecting job satisfaction, staff retention, and patient outcomes. The current study has demonstrated that anaesthetic nurses experience moral distress. Competent leadership, particularly in moral and ethical decision-making, is essential for effective resolution [50]. In a systematic review by Alluhaybi [51], examining nurse managers’ leadership, it was clearly stated that transformational leadership is considered the most effective style in nursing, balancing the organisation’s goals with the needs of nursing staff, eliminating transactional nursing leadership (leadership for pay check), and transitioning to the relational model, ensuring success for the organisation and fostering well-being for the nursing staff. Leaders who use this approach engage their teams by setting a positive example and giving each staff member personal attention, thereby increasing productivity [51]. Another example is a study in Kenya in which nurses in leadership positions struggle to balance the organisation’s needs with those of the nursing staff [52]. Adopting transformational leadership demonstrates that it effectively balances the needs of staff nurses with organisational objectives [52].

The institutional landscape, including professional bodies, legislation, and standards frameworks, represents another barrier to advancing anaesthetic nursing practice. Institutional constraints may be closely linked to hierarchical and leadership limitations, restricting formal recognition of anaesthetic nursing expertise. Anaesthetic nurses noted a gap between the realities of clinical work and the guidance offered by current regulatory standards. Existing anaesthetic nursing frameworks within Australia were seen as being limited in detail, and it was felt by the participants that clarity was needed to support and protect the specialised role of anaesthetic nurses. Without a dedicated legislative or a clearly defined professional framework, the expertise of anaesthetic nurses remains unclear, and their ability to practise at a full scope remains undefined [5]. Institutional constraints appear closely linked to hierarchical and leadership limitations, restricting formal recognition of anaesthetic nursing expertise. Without consistent standards and formal recognition, such as those seen in the United States, Sweden, and the Netherlands, anaesthetic nursing is likely to remain undervalued within Australia [5].

### 4.1. Implications for Practice

Drawing on the concerns, experiences, and aspirations articulated by nurses, this section outlines four key areas for consideration that emerged from the data. These areas are not presented as prescriptive or evidence-based recommendations, but as nurse-informed priorities reflecting perceived gaps and needs within current anaesthetic nursing practice. As such, they highlight issues that warrant further dialogue, policy attention, and future research.

Nurses described gaps in anaesthetic nursing practice that centred on limited career progression and fragmented educational pathways. These concerns were frequently linked to anxieties about the future sustainability and professional recognition of anaesthetic nursing roles within Australia. Some nurses expressed concern that, in the absence of clearer pathways and support, health services may increasingly rely on alternative workforce models, such as anaesthetic technicians or Operating Department Practitioners, as observed in other healthcare systems [53]. Nurses viewed such shifts as potentially influencing the scope, autonomy, and holistic clinical judgement traditionally associated with registered nursing roles.

In this context, four key nurse-informed areas for consideration are outlined, reflecting how anaesthetic nurses conceptualised the supports and conditions they believe are necessary to strengthen and sustain anaesthetic nursing practice in Australia and comparable health systems:

- Career pathway: Nurses consistently describe a perceived need for clearer career pathways and structured competency development to support progression within anaesthetic nursing.
- Education: Nurses expressed concern about fragmented educational preparation and highlighted the importance of more consistent and context-specific education aligned with anaesthetic practice.
- Leadership in nursing: Nurses emphasised the importance of supportive and visible leadership, particularly leadership that advocates for anaesthetic nursing roles and addresses ongoing hierarchical barriers.
- Legislation, standards, and professional bodies: Nurses identified a lack of clear standards and professional recognition as limiting role development, suggesting the need for greater institutional and regulatory attention.

Together, these nurse-informed priorities illustrate how anaesthetic nurses conceptualise the conditions required to support role development and provide a foundation for future empirical evaluation and policy deliberation.

### 4.2. Study Limitations

This study included 31 participants; however, the sample may not represent the full range of experiences among registered nurses in anaesthetic nursing across Australia, given the limited sample size and the fact that more than 50% were working in a single state. This limits the generalisability of the findings. In addition, the use of purposive and snowball sampling through professional networks may have introduced selection bias, as participants were likely to be more professionally engaged or motivated than those who did not participate. A further limitation concerns the data collection methods for a small number of participants who declined audio recording due to concerns about potential professional repercussions. While comprehensive notes were taken to preserve the integrity of these interviews, the absence of audio recordings may have limited the capture of nuance, emphasis, and exact phrasing. Importantly, participants’ reluctance to be recorded reflects broader issues of perceived vulnerability and power imbalance within anaesthetic nursing practice, which are central to the study’s findings. As such, this limitation also reinforces the contextual realities influencing nurses’ willingness to speak openly about their experiences.

Consistent with qualitative research more broadly, the aim of this study was not to achieve demographic or geographic representativeness but to generate an in-depth understanding of participants’ experiences. Accordingly, the findings should be interpreted as exploratory and illustrative, offering insight into common themes that may inform future, larger-scale research. Additionally, conducting interviews via videoconferencing may have introduced challenges, including the absence of in-person interaction, which may have limited the sharing of highly personal or sensitive information. While these findings do not claim to represent all anaesthetic nurses in Australia, they provide important exploratory insight into the challenges shaping anaesthetic nursing practice and identify areas requiring further investigation and policy attention.

Lastly, as with all qualitative research, data interpretation was shaped by the perspectives of multiple researchers. While reflexivity, peer debriefing, and collaborative analysis were used to enhance interpretive rigour, the possibility of interpretive bias cannot be eliminated entirely and should be considered when interpreting the findings.

## 5. Conclusions

This study explored the lived experiences of anaesthetic nurses in Australia and identified how culture, education, leadership, and institutional structures interact to shape everyday practice. Rather than operating as isolated influences, the findings suggest that these domains are interconnected and collectively structured by entrenched medical hierarchies that limit professional autonomy, visibility, and development. Across nurses’ accounts, hierarchical power relations emerged as a central mechanism influencing workplace culture, access to specialised education, leadership advocacy, and institutional recognition. These interrelated constraints contributed to anaesthetic nurses’ perceptions of being undervalued, restricted in career progression, and uncertain about the sustainability of their roles. Educational gaps, leadership challenges, and insufficient professional standards were not experienced as discrete problems but rather as mutually reinforcing factors that perpetuate the marginal positioning of anaesthetic nursing within perioperative care. Viewed holistically, the findings indicate that addressing single issues in isolation, such as education or leadership, may be insufficient without concurrent attention to broader cultural and structural conditions.

Strengthening anaesthetic nursing practice, therefore, requires systemic approaches that address hierarchical power dynamics, support advanced and standardised education pathways, foster visible and advocacy-focused nursing leadership, and enhance institutional recognition of anaesthetic nursing expertise. By articulating these relationships, this study contributes a more integrated understanding of the structural conditions shaping anaesthetic nursing in Australia. Supporting anaesthetic nurses through coherent, system-level reform has the potential not only to improve workforce sustainability but also to enhance the quality of anaesthetic care delivery.

While the findings offer important insight, they should be interpreted in light of the limitations of the study, including the dominance of participants from Queensland, which may reduce transferability to the broader Australian context, while a small number of interviews were conducted without audio recording, which may have led to a loss of accuracy of data. Lastly, the absence of anaesthetists’ perspectives highlights a key direction for future research.

## Figures and Tables

**Table 1:** Demographic information.

	Number (n = 31)	Percentage (%)
Gender		
Male	7	22.7%
Female	24	77.3%
Geographical Location		
Australian Capital Territory	1	3.3%
New South Wales	1	3.3%
Queensland	17	54.8%
South Australia	4	12.9%
Tasmania	1	3.2%
Victoria	5	16.1%
Western Australia	2	6.4%
Employment sector		
Public Hospitals	8	25.7%
Private Hospitals	16	51.6%
Both Public and Private Hospitals	6	19.5%
Prefer not to say	1	3.2%
Length of experience		
1 Year–3 Years	5	16.0%
4 Years–6 Years	4	12.9%
7 Years–10 Years	6	19.4%
10 Years–20 Years	10	32.3%
20 Years Above	6	19.4%
Educational background above a bachelor’s degree		
Master’s Degree	7	22.5%
Grad Certificate or Graduate Diploma	14	45.2%
Peri-Operative Graduate Programme (Transition support programme to peri-operative nursing)	2	6.5%
On-the-job training (Provided by the health service)	3	9.7%
Did not wish to disclose	5	16.1%

## Data Availability

The data presented in this study are available on request from the corresponding author due to ethical reasons.

## Public Involvement Statement

No public involvement in any aspect of this research.

## Guidelines and Standards Statement

This manuscript was drafted against the Consolidated Criteria for Reporting Qualitative Research (COREQ) guidelines for Qualitative Research.

## Appendix A

Interview questions

1. Tell me about your nursing story. For example, how long have you been in anaesthetic nursing, and how did you get into this specialty?
2. Which pathway did you take to get your certification or training in anaesthetic nursing?
3. How has your role evolved over time?
4. What parts of anaesthetic nursing are most rewarding? What parts are the most challenging?
5. If you could change or improve any aspect of anaesthetic nursing practice, what would it be?
6. What are some of the obstacles that stop you from performing your role?
7. What are your thoughts regarding the current scope of practice for anaesthetic nurses in Australia and on expanding the role of anaesthetic nurses, such as advancing into nurse sedationist or nurse practitioner roles?
8. What additional training or support might be necessary if the scope of nursing practice were expanded?
9. Do you have anaesthetic technicians in your clinical setting? What are your thoughts about having anaesthetic technicians, and how does or would their role impact your practice?
